# Predicting Curing Distortion in Composite Manufacturing—A Fast and Cost-Efficient Numerical Simulation Method

**DOI:** 10.3390/polym16243597

**Published:** 2024-12-23

**Authors:** Yongming Zhang, Luling An, Cong Zhao

**Affiliations:** Collage of Mechanical & Electrical Engineering, Nanjing University of Aeronautics and Astronautics, Nanjing 210016, China; zhangym@nuaa.edu.cn

**Keywords:** polymer matrix composites, warping, sequential coupling simulation method, Ω-shaped composite laminates, FEM

## Abstract

The curing distortion is a critical determinant of the quality of integrally manufactured composite structures, playing a pivotal role in the design and fabrication of composite. This paper presents two simplified methods in predicting the curing distortion for large-scale composite aircraft structures manufactured through the autoclave process. Firstly, the refined finite element models of the two simplified methods were developed. Then, it was utilized to predict the curing distortion of Ω-shaped composite laminates. The comparative study between the experimental data and numerical results shows that the proposed second simplified method balanced the prediction accuracy and efficiency, which is urgently needed in practice. Finally, using the second simplified method, predictions were conducted for the curing distortion of practical large-scale composite skin structures. The results were in good agreement with the corresponding experiments. This study provides a new solution for the rapid iterative design of large-scale composite structures, as well as the efficient design of frame molds for their manufacturing.

## 1. Introduction

Due to high specific strength and specific stiffness, polymer matrix composites become attractive alternatives for a wide range of high-performance applications [1]. The utilization of integrated technology for the forming of composite structures can significantly reduce the number of parts and connectors, enhance the reliability of composite material structures, and lower production costs [2]. Currently, the autoclave process is the primary technology employed for the integral molding of thermosetting resin-based composites [3,4]. During the molding process utilizing the autoclave technique, heating operations play a crucial role in elevating the ambient temperature surrounding the composite material, effectively initiating the curing process of the resin. Throughout the entire molding process, the resin undergoes a sequence of intricate physical and chemical transformations, leading to notable modifications in the material’s state. After demolding, composite structures typically exhibit curing distortion phenomena [5,6]. Excessive curing distortion can present significant challenges during the assembly of composites, potentially resulting in assembly failures or the scrapping of components due to exceeding allowable tolerances [5,7]. Therefore, curing distortion is a crucial factor that impacts the quality of integrally formed composite structures, and it remains one of the key issues in the design and manufacturing of composite materials. To effectively reduce curing distortion in composite materials, traditional techniques involve meticulous iterative adjustments and compensatory designs to optimize curing process curves and mold configurations, drawing upon extensive empirical knowledge and rigorous process experimentation in the realm of technical expertise. However, for composite materials with intricate shapes or composed of novel component materials, the aforementioned empirical knowledge may not be applicable, and the efficiency of experimental iteration can be relatively low. Accurate and fast prediction of curing distortion in composite structures can effectively enhance the efficiency of mold design and reduce costs.

At present, numerical simulation methods are the most commonly used predictive methods [5,8,9,10,11]. During the curing process of composite materials, they transition through three distinct material states: viscous flow, rubbery, and glassy. The primary focus of numerical simulations lies in accurately capturing and modeling the intricate series of physicochemical processes occurring during these transitional stages. Therefore, researchers focus on the characterization of constitutive behavior of resin matrix in the curing process. Now, the widely used existing constitutive models of resin are mainly two types: the viscoelastic model [12,13] and elastic model [14,15]. The viscoelastic model represents more detailed mechanisms, but the equations are complex, and the calculation is time-consuming. To reduce the computational time, researchers proposed some simplified viscoelastic models [16,17]. These simplified viscoelastic models achieve reduced computation time while inevitably compromising on the level of predictive accuracy. However, reducing the solution time by sacrificing accuracy is not the result we desire. On the other hand, there is still room for improvement to decrease the computing cost.

**Figure 1:**
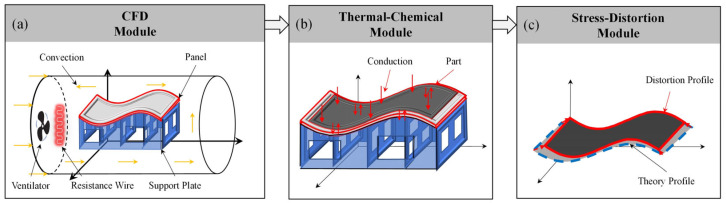
Sequential coupling analysis method.

Another significant factor that affects the computation time of numerical simulation methods is the amount of data in the numerical model. Under the same conditions of solving resources, the more grid elements in the grid model, the longer the solving time will be. Currently, the numerical simulation method for the composite curing distortion is a sequential coupling analysis (SCA) method. The numerical model for the SCA method comprises three distinct modules: a computational fluid dynamics (CFD) module, a thermal-chemical module, and a stress-displacement module, as illustrated in Figure 1.

In the first phase, the CFD module is employed to precisely determine the temperature field distribution within the framed mold, as exemplified in Figure 1a. Firstly, geometric models for the fluid domain and solid domain are established. Subsequently, mesh models for the autoclave and the framed mold are constructed. Based on the temperature curing process curve, the inlet temperature load and fan rotation speed are set for the mesh model of the autoclave. Dynamic flow field analysis is then conducted on the framed mold for composite material forming by heating the internal air within the autoclave.

In the next phase, the nodal temperatures of the framed mold are imported into the thermal-chemical module. Boundary conditions and loads are then set to calculate the temperature field and degree of cure field of the composite structures, as shown in Figure 1b.

In the final phase, the precisely calculated temperature field and degree of cure field data for the composite structures obtained in the second step are imported into the stress-deformation analysis module to thoroughly simulate and compute the curing distortion of the composite structures during the curing process, as depicted in Figure 1c.

However, composite structures exhibit a trend towards integrated and large-scale development. The continued reliance on the numerical model developed via the SCA method, characterized by a colossal number of grid elements, substantially impedes the efficiency of the solution process. In particular, the number of grid elements in the mesh model within the CFD module is enormous, significantly compromising the efficiency of the solution process. For instance, in a Φ3 × 10 m autoclave model, the number of grid elements in both the fluid domain and solid domain can reach tens of millions or even more. The extensive number of grid elements poses a significant impediment to the efficiency of the solution process. Furthermore, within the thermal-chemical module, it is imperative to incorporate the temperature field data of the entire framed mold’s grid nodes, serving as critical temperature loads for the curing process of the composite material component. Within the thermal-chemical module, the influence of the temperature field of the framed mold, encompassing both the overall mold temperature profile and the specific temperature distribution of the mold panel in direct contact with the composite material component, is negligible in determining the temperature distribution within the composite itself.

In conclusion, the present study introduces a refined simplification method and constructs a numerical simulation model, with the view of minimizing the data volume required for anticipating the warping of composite structures. This method effectively streamlines the computation process while preserving the predictive accuracy, thereby enhancing the overall efficiency of the numerical analysis. In this paper, we first discuss the composition of the numerical model for the SCA method, along with the results obtained from each individual component of the model. Based on this discussion, two simplification methods are proposed, and numerical models for these methods are established accordingly. The superiority of the proposed simplification methods is demonstrated through comparisons with the results obtained from the existing SCA methods. Afterwards, Ω-shaped composite laminates and a large-scale composite structure were fabricated. Finally, their warping was measured and compared with the FEM results.

## 2. A Fast and Cost-Efficient Numerical Simulation Method for Prediction Curing Distortion of Composite

### 2.1. Simplified Method—Retention of the Mold Panel Temperature Field in a Thermal-Chemical Module

**Figure 2:**
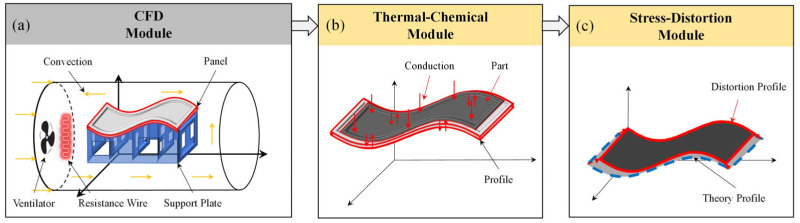
The numerical simulation model refined by the first simplification method.

The specific objective of the first simplified method is to streamline the numerical model of the thermal-chemical module by reducing the number of grid cells, thereby enabling more efficient computations and analyses. Compared to the existing SCA methods, the present method introduces innovations within the thermal-chemical module. Specifically, we solely incorporate the temperature field data of the mold panel into this module and restrict the participation in the solution process solely to the mold panel. Given the successful utilization of the CFD module in deriving the temperature field distribution across the grid nodes of the comprehensive framed mold, the temperature values corresponding to each grid node have been comprehensively archived within the designated temperature field, as shown in Figure 2a.

In the CFD module, the overall mold is divided into two parts: the mold support plate and the mold panel, with temperature fields obtained for each part. During the curing process, the primary heat source for the component is heat transfer. Therefore, in the thermal-chemical module, the temperature field of the mold support plate does not influence the prediction of the component’s temperature field and degree of cure; only the temperature field of the mold panel, which is in direct contact with the component, is relevant. Therefore, within the thermal-chemical module, the sole integration of temperature field data pertaining to the grid nodes of the mold panel does not exert a significant influence on the precision of curing distortion predictions, as shown in Figure 2b. The stress-deformation module utilizing the first simplified method is depicted in Figure 2c. This model is consistent with the SCA method. After implementing the first simplified method, we successfully developed a numerical simulation model that accurately predicts the curing distortion of composite laminates.

### 2.2. Simplified Method—Elimination of the CFD Module and Support Plate Geometry

The second simplification method’s underlying principle involves optimizing the grid density of the numerical simulation models within both the CFD module and the thermal-chemical module, ultimately leading to improved computational efficiency. Given the relatively gradual heat-up rate within the autoclave, coupled with the existence of the frame structure, the surface temperature distribution of the framed mold exhibits a nearly uniform pattern. Furthermore, even in the heat-up stage, the presence of a certain temperature difference results in a relatively low residual stress in the component due to the low Young’s modulus of the resin. Based on this phenomenon, we assume that the temperature on the outer surface of the mold remains uniform, and the heat-up rate is consistent throughout the solidification process. Therefore, we utilize the theoretical process temperature as a substitute for the temperature field of the framed mold derived from the CFD module. Hence, we opt to employ the theoretical process temperature as a replacement for the temperature field of the framed mold, as obtained from the CFD module. Additionally, building on the first simplification method, it can be concluded that the supporting plate can also be simplified.

**Figure 3:**
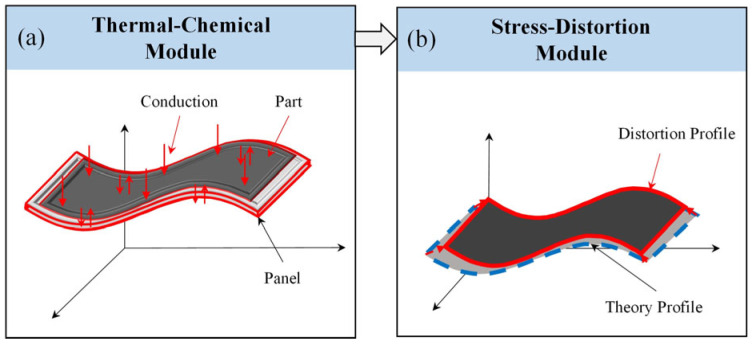
The numerical simulation model refined by the second simplification method.

In conclusion, our method differs significantly from the current sequential coupling analysis method, primarily due to the elimination of the CFD module and the subsequent simplification of the geometric model of the framed mold within the thermal-chemical module, as shown in Figure 3a. After implementing the second simplification method, we successfully developed a numerical simulation model that accurately predicts the solidification deformation of composite material components, as shown in Figure 3b.

## 3. The Curing Distortion of Ω-Shaped Composite Laminates

### 3.1. FEM Simulation

The finite element analysis software employed in this paper was Simcenter 3D (version: 1954). The material used in this study was ZT7H/QY9611 plain weave fabric prepreg provided by Nanjing Julong Composite Technology Co., Ltd. The fiber content accounted for around 67% of the weight. The mechanical properties and CTEs of ZT7H/QY9611 in glassy state were tested as shown in Table 1 [18,19,20]. The mechanical properties in rubbery state were estimated considering that the modulus of resin in rubbery state is nearly 1/100 of the one in glassy state [19]. The mechanical behavior of the resin is characterized using a viscoelastic constitutive model, as detailed in [20,21]. The mold material was composed of Q235-A steel, with associated performance parameters conforming to those reported in Ref. [18].

#### 3.1.1. Heat Transfer Model

In an autoclave, air is heated to control the temperature of the mold and the component during the curing process through solid heat transfer. The temperature transfer and distribution within composite components are regarded as a nonlinear endothermic problem. This is based on Fourier’s law of heat transfer and the law of conservation of energy, leading to the establishment of the following equation as the governing equation for heat transfer and distribution in an autoclave [22,23]:

(1) $$\frac{\partial }{\partial x}\left[k_{x}\frac{\partial T}{\partial x}\right]+\frac{\partial }{\partial y}\left[k_{y}\frac{\partial T}{\partial y}\right]+\frac{\partial }{\partial z}\left[k_{z}\frac{\partial T}{\partial z}\right]+\overset{\cdot }{Q}=C_{T}\frac{\partial T}{\partial t}$$

where *T* stands for temperature; *C_T_* represents the specific heat of the material; and *k_x_*, *k_y_*, and *k_z_* are the coefficients of heat transfer along the *x*, *y*, and *z* directions.

$\overset{\cdot }{Q}$ is the rate of heat generation related to the exothermic nature of the curing reaction and is expressed as the following equation [19]:

(2) $$\overset{\cdot }{Q}=\left(1-V_{f}\right)H_{r}\frac{d\alpha }{dt}$$

where *t* is time, *V_f_* is the fiber volume content, *H_r_* is the total exothermic reaction heat per unit mass of resin cured, and *α* is the degree of cure.

#### 3.1.2. Curing Kinetics Model

The reaction kinetics and glass transition temperature of QY9611 resin are described by [24].

(3) $$\frac{d\alpha }{dt}=2.28\times 10^{7}\exp\left(\frac{-11114.9}{T}\right){\left(1-\alpha \right)}^{1.485}$$

(4) $$T_{g}=-264.04+\frac{120.46\alpha }{1-0.5176\alpha }$$

**Table 1:** Properties of ZT7H/QY9611.

Property	Rubbery	Glassy
*E*_1_/GPa	140	144
*E*_2_ = *E*_3*f*_/GPa	0.16	10.2
*ν*_12_ = *ν*_13*f*_	0.3	0.3
*ν*_23_	0.6	0.4
*G*_12_ = *G*_13*f*_/GPa	0.05	6.0
*G*_23_/GPa	0.04	3.0
*α*_1_/(*με*·K^−1^)	0.2	
*α*_2_ = *α*_3_/(*με*·K^−1^)	40.9	
*β*_1_/*με* [23]	−167	
*β*_2_ = *β*_3_/*με* [23]	−8810	

#### 3.1.3. Finite Element Model of the First Simplified Method

**Figure 4:**
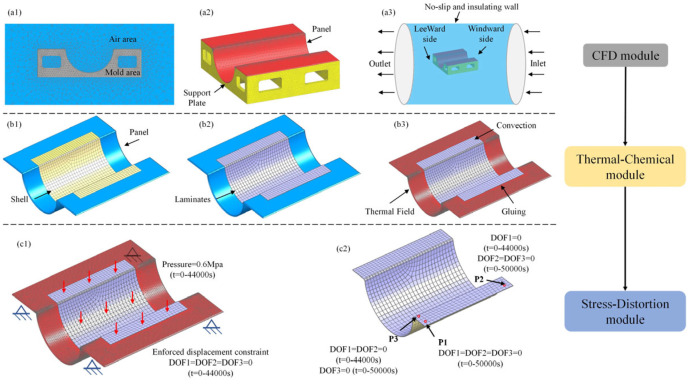
Analysis flowchart and FEM of the first simplified method.

Utilizing the numerical model proposed in Section 2.1 for predicting the curing distortion of composite laminates, we successfully established a numerical model for the Ω-shaped composite laminates, as depicted in Figure 4. An unstructured mesh was selected due to the complex mold structure. During the process of fluid–solid conjugated heat transfer, the mesh condition of the fluid–solid conjugated interface influenced the accuracy of the simulation, as shown in Figure 4(a1). The red area represents the mold panel structure, while the yellow area denotes the support plate structure, as shown in Figure 4(a2). The FEM of the CFD module is shown in Figure 4(a3). When the maximum grid size was reduced from 300 mm to 250 mm, the maximum temperature variation in the mold was 0.1 °C. Considering both the solution accuracy and efficiency, the maximum grid size was determined to be 300 mm. The model employs a C3D4 tetrahedral mesh with a maximum element size of 300 mm, comprising a total of 79,069 elements. The convective model is consistent with that reported in Ref. [18]. The inlet fluid velocity of the model was set at 3 m/s, with the inlet temperature profile following the manufacturer’s recommendations, and the outlet pressure was set at 0.01 MPa. The value of the initial temperature load for this FEM was 298K. The fluid–solid contact position was set as a no-slip interface boundary. The flow state of the gas was turbulent flow [18]. Utilizing the CFD module, we can obtain the temperature field distribution of both the mold panel structure and the support plate structure.

As depicted in Figure 4(b1), the mold panel structure was discretized into shell element meshes, adhering to the contour lines of the composite laminate. Subsequently, a three-dimensional model of the Ω-shaped composite laminate was constructed through a thickening operation utilizing the shell elements, as depicted in Figure 4(b2). The FEM of the thermal-chemical module is presented in Figure 4(b3). The DC3D8R solid element was used in the simulation as it can represent the mechanical behavior of the material properly in the thickness direction compared to shell elements [20]. When the maximum mesh size was adjusted from 20 mm to 15 mm, the maximum temperature difference in the composite laminate changed by only 0.01 °C. Considering both computational efficiency and accuracy, the final mesh size was determined to be 20 mm. The number of meshes was 201,553. The temperature load consisted of two sections. One section was the temperature field of the mold panel during the curing process obtained by the CFD module. The other section was the temperature load at the outer surface of the composite laminates exposed to air, also known as the process temperature. The upper surface of the mold and the under-surface of the composite laminates were set as surface-to-surface prefect gluing boundary conditions. Utilizing the thermal-chemical module, we were able to precisely determine the temperature field and degree of cure distribution within the Ω-shaped composite laminates.

The stress-displacement module incorporates two sub-analysis steps: one during the curing process and another demolding. The analysis step during the curing process refers to the forming stage of the composite laminate within the framed mold, as illustrated in Figure 4(c1). The demolding analysis step pertains to the stage after the composite laminate is separated from the framed mold, as depicted in Figure 4(c2).

The mesh type was C3D8, and the number of meshes was 201553. When the mesh size was adjusted from 20 mm to 15 mm, the maximum curing distortion of the composite laminate changed by only 0.01 mm. Considering both the accuracy and efficiency of the solution, the final maximum mesh size was determined to be 20 mm. Furthermore, the results obtained from the thermal-chemical model were linked to the stress-displacement module as the applied load. According to the curing process curve, a pressure load of 0.6 MPa was applied to the upper surface of the component. The interface friction coefficient between the mold and the structure was set to 0.5 [20,25]. To achieve the accuracy of the prediction of the demolding after the curing distortion of composite laminates, we proposed a three-point constraint method. First, the locations of the three constraint points were determined based on the measurement methods of the curing experiment. Next, the relative positions of the three-points were distributed at right angles. This was to ensure that the demolding stage of the composite laminates, the freedom of movement, and stress relief were not restricted, thereby affecting the accuracy of the prediction.

The boundary conditions included three sections. In the first section, the lower surface of the mold support plates was provided with an enforced displacement over time (starting from t = 0, the displacement value was 0 until the part was demolded). In the second section, the edges of the laminate were set as enforced displacements over time, similarly (start from t = 0, the displacement value was 0 until the part was demolded). In the third section, after the demolded stage, it was necessary to set the constraints of the demolded composite laminates. This condition is called a three-point constraint. The basic principle of the three-point constraint is that the three-points are in a plane and form a right triangle. All three degrees of freedom for the first point are constrained. One of the degrees of freedom of the second point is constrained until demolded, while the other two degrees of freedom are all constrained. Two of the degrees of freedom at the third point are constrained until demolded, and the other is constrained in all degrees of freedom. During the curing process, the tangential behavior between the mold/parts is set as the classical type of friction.

#### 3.1.4. Finite Element Model of the Second Simplified Method

**Figure 5:**
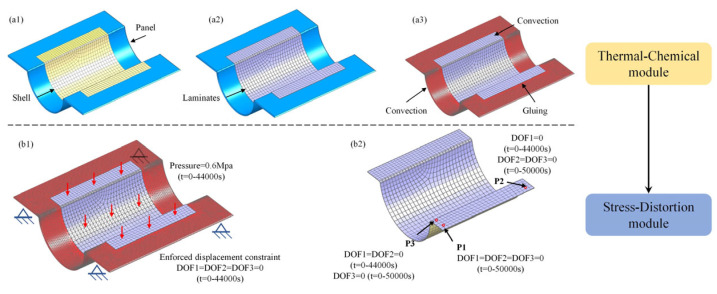
Analysis flowchart and FEM of the second simplified method.

Utilizing the numerical model proposed in Section 2.2 for predicting the curing distortion of composite laminates, we successfully established a numerical model for the Ω-shaped composite laminates, as depicted in Figure 5. In the FEM of the thermal-chemical module, the load solely comprised temperature convection, as shown in Figure 5(a1–a3). All other boundary conditions and loads remained consistent with the FEM of the first simplified method, as shown in Figure 5(b1,b2).

### 3.2. Experiment

**Figure 6:**
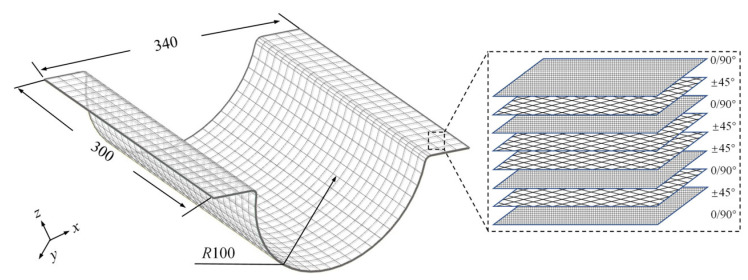
Ω-shaped composite laminates.

**Figure 7:**
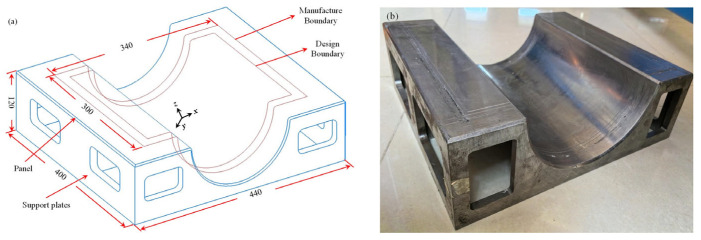
Framed mold.

Figure 6 comprehensively illustrates the specific shape, dimensions, and stacking configuration of the Ω-shaped composite laminates, providing a comprehensive overview of their structural characteristics. Figure 7a,b demonstrate the shape and dimensional design of the framed mold utilized for testing purposes. The framed mold had dimensions of 440 × 400 × 120 mm, with a panel thickness amounting to 5.5 mm. The rectangular ventilation holes, characterized by chamfered edges, were evenly spaced across both the panel and the support plates.

Firstly, the prepreg was laid on the mold panel according to the stacking sequence [0/45/0/45/45/0/45/0]_s_ with a thickness of 0.125 mm for the single-layer prepreg. Then, we put the barrier film and breathable felt on the laminates in order. We then sealed the vacuum bag with putty strips on the mold. The curing process curve consisted of two thermostatic processes. The temperature was heated up to 393.15 K at a rate of 275.15 K·min^−1^ and kept for 60 min. Then, the temperature was increased to 453.15 K at 274.15 K·min^−1^ and kept for 380 min. Finally, it was cooled down uniformly to room temperature at 274.29 K·min^−1^.

## 4. Results and Discussion

### 4.1. Experimental and Simulation Results

**Figure 8:**
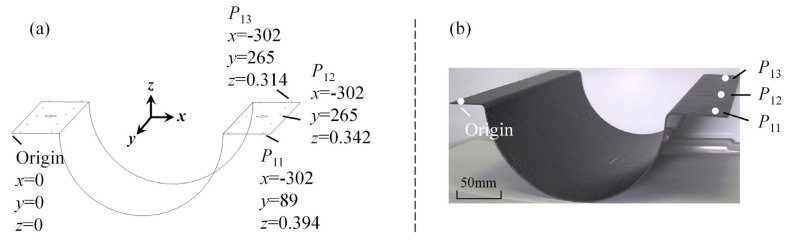
The cure distortion experimental samples and measurement results.

Using a coordinate measuring machine (CMM), the relative coordinates of some point’s structure were measured on the composite. The *z*-distance between the coordinates of the measuring point and the coordinates of the origin was the cured distortion. We selected three groups of experimental laminates for measurement. The two positions on the right side were randomly selected to measure the amount of curing distortion, the exact measurement means, and the process, as shown in Figure 8a. The physical image of the cured Ω-shaped composite laminate is shown in Figure 8b.

**Figure 9:**
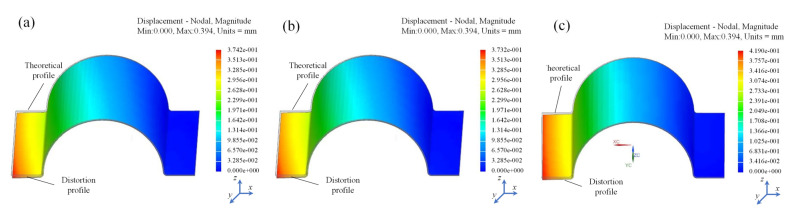
The simplification-method-specific predicted curing distortion.

The curing distortion of the Ω-shaped cross-ply composite laminates was employed to corroborate the superiority of the proposed methods. Based on the FEMs established in Section 3.1.1 and Section 3.1.2, the curing distortion results of the Ω-shaped composite laminates were obtained. The predicted curing distortion results of the SCA method, the first simplified method, and the second simplified method are presented in Figure 9a–c, respectively. The figure includes both the theoretical surface and the deformed surface of the Ω-shaped composite laminates. The predicted amplitudes of curing distortion in composite laminates—utilizing the SCA method, the first simplified method, and the second simplified method—were 0.374 mm, 0.373 mm, and 0.419 mm, respectively.

The measured curing distortion in the experiment and the predicted ones in the FE model are shown in Table 2. The detailed narrations and discussions are shown in the next section. Three types of solving methods, that is, the SCA method, the first simplified method, and the second simplified method, were used to predict the curing distortion. There still was small deviations between the experimental and predicted results. The reasons may be measurement errors in the experiment or input parameter errors in the simulation.

### 4.2. Comparison of Curing Distortions Calculated by the Proposed Methods and Existing Method

**Figure 10:**
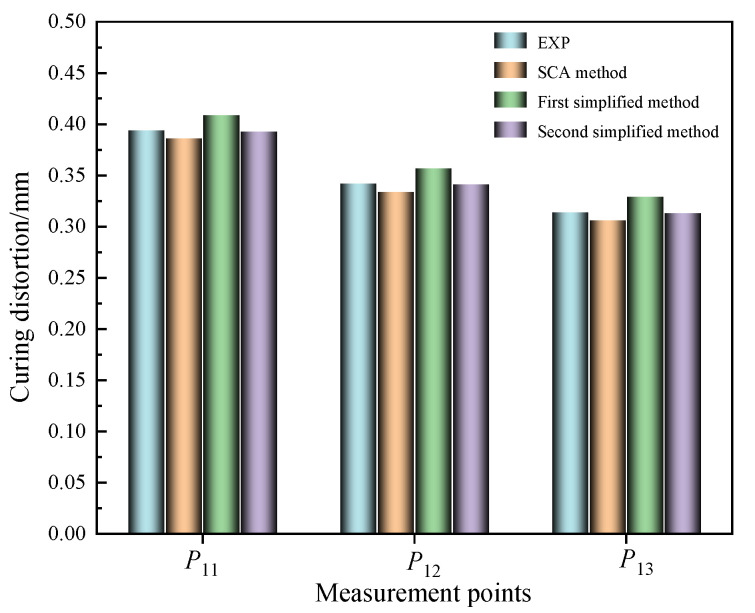
Comparison of curing distortion prediction for different methods.

The comparison of the experiment (EXP) results with the FEM results of the curing distortion of the SCA method and two simplified methods for the Ω-shaped composite laminates was as shown in Figure 10. Here, we analyze and discuss the results of curing distortion from three prediction methods based on the data presented in Table 2.

In comparison with the results of curing experiments, the SCA method and the first simplified method demonstrated relatively small prediction errors. However, the second simplified method exhibited a comparatively larger prediction error. Both the SCA method and the second simplified method had prediction errors within 15%.

When comparing the prediction accuracy of different methods for curing distortion, we observed variations in their performance across different measurement points. Taking the *P*_11_ point as an example, the SCA method exhibited a prediction error of 5.1% compared to the EXP data, while the first simplified method had an error of 5.3%. In contrast, the second simplified method displayed relatively lower prediction accuracy, with an error of 6.3%. Similarly, at the *P*_12_ point, the SCA method’s prediction error reached 6.4%, and the first simplified method had an error of 6.5%. In contrast, the second simplified method displayed relatively lower prediction accuracy, with an error of 11.7%. For the *P*_13_ point, the SCA method’s prediction error was 4.2%, and the first simplified method’s error was 4.6%. In contrast, the second simplified method displayed relatively lower prediction accuracy, with an error of 8.6%.

From the error analysis of the aforementioned three points, it is evident that the first simplified method exhibited relatively high accuracy in predicting the curing distortion of composite laminates. The prediction error of the first simplified method was generally consistent with that of the SCA method. However, compared to the SCA method, the prediction error of the second simplified method was relatively larger, with the maximum prediction error reaching 11.7%. Nevertheless, the prediction errors of the second simplified method were all around 10%, which still demonstrated a relatively high prediction accuracy.

**Figure 11:**
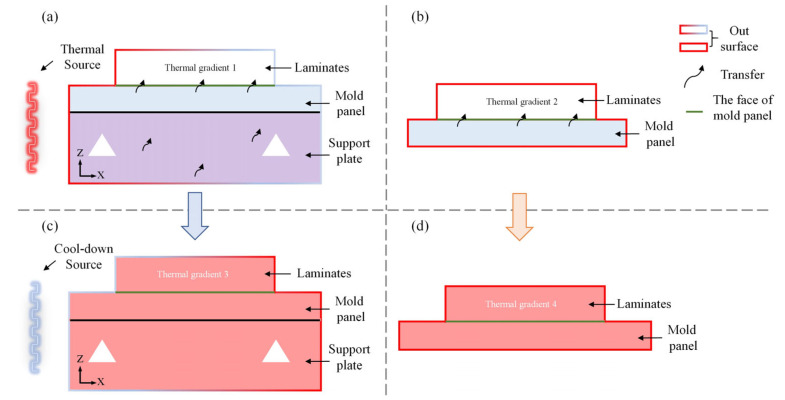
Thermal gradient for different methods.

As is well known, the distortion of composite structures during curing is most directly attributed to the accumulation of residual stress. Therefore, accurately capturing the distribution of residual stress becomes crucial. As depicted in Figure 11a, the CFD module within the SCA method and the first simplified method revealed the thermal distribution on the outer surface of the framed mold and the composite laminates during the heat-up stage. Under the influence of the heat source, the thermal in areas close to the heat source was significantly higher than those far away, resulting in the formation of thermal gradient 1 along the thickness direction (z-direction) of the composite laminates, which subsequently led to the generation of residual stress. However, as illustrated in Figure 11b, based on the fundamental assumption of the second simplified method, although the outer surface thermal of the composite laminates was deemed uniform, a thermal gradient 2 still formed in reality, also contributing to the accumulation of residual stress. It is noteworthy that in the second simplified method, the average thermal on the out surface of the composite laminates was similar to the high-thermal region in Figure 11a. Consequently, the thermal gradient 2 formed in this method was greater than the thermal gradient 1 in the SCA method, potentially leading to an overestimation of the curing distortion prediction. However, the degree of this overestimation was not significant, as existing research indicates that the accumulation of residual stress during the heat-up stage is relatively small, resulting in minimal differences in the prediction results compared to the SCA method.

During the keeping temperature stage, the overall temperature tended to become uniform. When the cool-down stage began, the initial conditions remained consistent regardless of whether the SCA method or the second simplified method was used. As the thermal of the out surface of the composite laminates was lower than the internal thermal during the cool-down process, a thermal gradient was once again formed, leading to the generation of residual stress, as shown in Figure 11c,d. In Figure 11c, the surface temperature near the cool-down source was lower than the surface far from it, resulting in a thermal gradient 3 that was smaller compared to the thermal gradient 4 in Figure 11d (the second simplified method). Based on the above analysis, the SCA method and the first simplified method had similar errors in predicting curing distortion. However, due to the larger thermal gradient generated by the second simplified method, its prediction of curing distortion tended to be higher.

### 4.3. Comparison of Prediction Efficiency by the Proposed Methods and Existing Method

**Figure 12:**
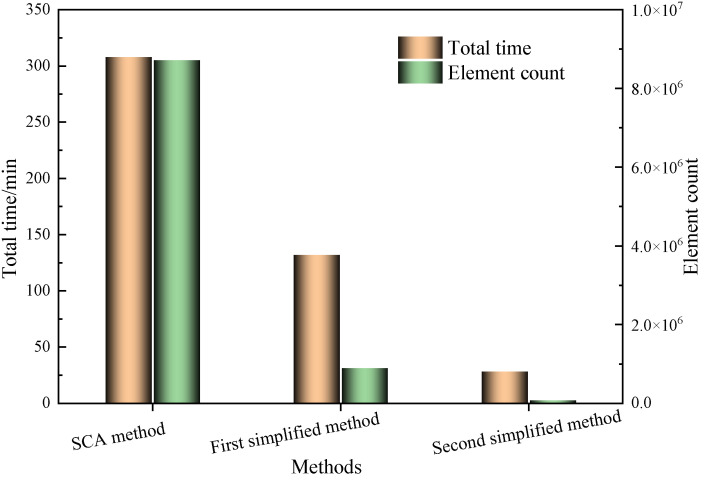
Comparison of total time and element count prediction for different solution methods.

The computer configuration used for the simulation calculations was an Intel i5-10400@2.90 GHz six-core/12-thread CPU with 128 GB of RAM. The total calculation time for the SCA method and the different solute method is shown in Figure 12. The total calculation time of the SCA method was 308 min. The total calculation times for the first and second simplified method were 132 min and 28.2 min, respectively. From a holistic view of solution time optimization, the first simplified method notably decreased the computation time by 57.1% in comparison to the traditional SCA method, whereas the second simplified method exhibited an exceptional reduction of 90.8%. Upon a closer analysis of the comparative data on element count, it is apparent that the traditional SCA method employed 8,714,764 elements, whereas the first simplified method trimmed it down to 895,168 elements, marking a substantial drop of 89.7%. However, the second simplified method stood as a testament to the extreme simplified method, utilizing only 75,906 elements, a mind-boggling 99.1% reduction from the traditional SCA method.

Aligning with the insights from the thorough study in Section 4.1, we can confidently assert that the second simplified method not only maintains the precision in predicting the curing distortion of composite laminates but also significantly enhances computational efficiency, achieving a harmonious equilibrium between accuracy and efficiency. Therefore, it is recommended to utilize the second simplified method for predicting curing distortion in relevant research or engineering applications to ensure the accuracy and reliability of the results.

## 5. Prediction of Curing Distortion of Practical Composite Skin Based on the Method Proposed in This Paper

In Section 3, the two simplified methods were investigated with relatively simple Ω-shaped composite laminates. In this section, the rationality and efficiency of the second simplified method are verified for a practical composite skin. In engineering, composite structures have larger scales; higher complexity in shape and layer information; and will experience rebound distortion after demolding, which is difficult to predict. We took a certain type of aviation composite structure as an example for numerical verification.

The aircraft composite structures had a complex shape and significant curvature variation. The overall size was approximately 3000 × 2000 mm. The composite structures had complex stacking sequence information, with nearly 100 layers inside. The thickness of structures varied at different positions and included the area of layer loss. The type of carbon fiber epoxy resin prepreg and the mold material should be consistent with those selected for the forming of Ω-shaped composite laminates.

**Figure 13:**
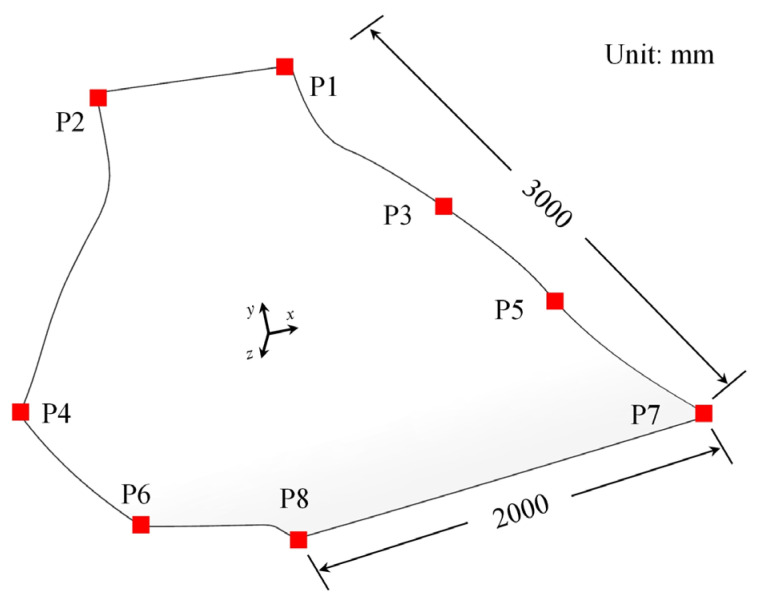
Location distribution of measurement points.

**Figure 14:**
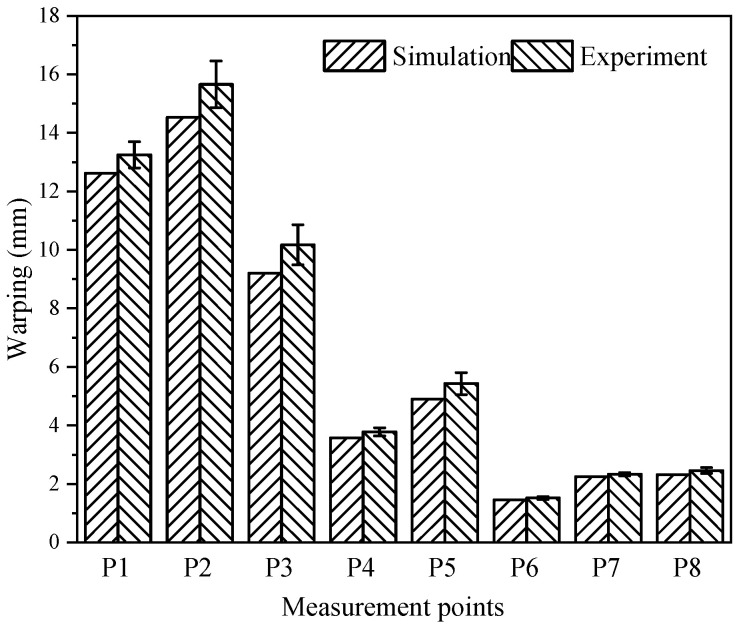
Comparison of simulation and experiment results.

We applied the second simplified method to predict curing distortion composite structures in the aircraft field. The curing process curve was consistent with the previous section. Measure the curing distortion results for positions 1~8 of the composite structures was performed as shown in Figure 13. The curvature of the upper end of the structure changed significantly. Its thickness was uneven, and the sharp corners were sloping. Therefore, we fixed the lower plane during measurement. This led to large distortion at measurement points 1/2/3, and the distortion at the lower measurement point 6/7/8 was relatively small. The curing distortion prediction results of the second simplified method were compared with the experimental results, as shown in Figure 14.

Comparing the final curing distortion, we can find that the second simplified method proposed a better match with the experimental results. The solution efficiency was significantly improved. The location and size of the maximum curing distortion of the composite structures were uniform, using both the second simplified method. Two aspects caused this phenomenon: firstly, the thermal gradient of the overall surface had less influence on the results, whether it was different structure dimensions and different layups affecting the composite material. Secondly, due to the existence of an edge difference between the composite structure and the molded panel, the temperature loads received at the edge difference were complex, resulting in a larger internal stress at the edge of the structure during the curing process than at other locations, which caused a larger curing distortion. The curing distortion of composite structures can be improved by appropriately enlarging the manufacturing boundaries of the composite structures, or by reducing the boundary distances.

## 6. Conclusions

In this paper, two simplified methods were proposed for the fast and cost-efficient prediction of curing distortion based on the analysis of the existing SCA methods. The advantages of the proposed model are shown by being compared with the existing models. The process of establishing the FEM for predicting the curing distortion of composite structures was analyzed in detail. Then, the curing distortion of Ω-shaped composite laminates and large-scale composite skin structures were investigated based on the proposed simplification method. Finally, the present study undertook a comprehensive large-scale curing experiment to rigorously assess the predictive accuracy of the two simplified methodologies in relation to curing distortion. Some conclusions can be extracted from the results and discussions:

(1) The simulation accuracy of the curing distortion of composite material structures using the second simplified method proposed was approximately the same as that of the SCA method, but the computational efficiency was greatly improved. The proposed simplified method balanced the simulation accuracy and efficiency, which is urgently needed in practice.
(2) Utilizing the second simplified method, we accurately predicted the curing distortion of large-scale composite structures and conducted a detailed comparison with experimental data obtained from eight points during curing experiments. The maximum prediction error was only 9.8%, and the prediction efficiency was significantly improved.
(3) This study provides a new solution for the rapid iterative design of large-scale composite structures, as well as the efficient design of frame molds for their manufacturing.

## Figures and Tables

**Table 2:** The measured and predicted curing distortion of samples.

Points	EXP	Predicted by the SCA Method	Predicted by the First Simplified Method	Predicted by the Second Simplified Method
*P*_11_	0.394	0.374	0.373	0.419
*P*_12_	0.342	0.320	0.321	0.382
*P*_13_	0.314	0.301	0.300	0.341

## Data Availability

The raw data supporting the conclusions of this article will be made available by the authors on request.
